# Wash-in and wash-out of sevoflurane in a test-lung model: A comparison between Aisys and FLOW-i

**DOI:** 10.12688/f1000research.11255.2

**Published:** 2017-04-26

**Authors:** Petter Jakobsson, Madleine Lindgren, Jan G. Jakobsson

**Affiliations:** 1 Faculty of Medicine, University of Lund, Lund, 221 00, Sweden; 2 Department of Anaesthesia & Intensive Care, Institution for Clinical Science, Karolinska Institutet, Danderyds University Hospital, Stockholm, 182 88 , Sweden

**Keywords:** wash-in, low-flow anaesthesia, MAC, End-tidal concentration, sevoflurane

## Abstract

**Background:** Modern anaesthesia workstations are reassuringly tight and are equipped with effective gas monitoring, thus providing good opportunities for low/minimal flow anaesthesia. A prerequisite for effective low flow anaesthesia is the possibility to rapidly increase and decrease gas concentrations in the circle system, thereby controlling the depth of anaesthesia. **Methods:** We studied the wash-in and wash-out of sevoflurane in the circle system with fixed fresh gas flow and vaporizer setting. We compared two modern anaesthesia work stations, the Aisys (GE, Madison, WI, USA) and FLOW-i (Maquet, Solna, Sweden) in a test lung model. **Results**: We found fresh-gas flow to have, as expected, a major influence on wash-in, as well as wash-out of sevoflurane. The wash-in time to reach a stable circle 1 MAC (2.1%) decreased from an average of 547 ± 83 seconds with a constant fresh gas flow of 300 ml/min and vaporizer setting of 8%, to a mean of 38 ± 6 seconds at a fresh gas flow of 4 L/min. There were only minor differences between the two works-stations tested; the Aisys was slightly faster at both 300 and 4 L/min flow. Time to further increase circle end-tidal concentration from 1-1.5 MAC showed likewise significant associations to fresh gas and decreased from 330 ± 24 seconds at 300 ml/min. to less than a minute at constant 4 L/min (17 ± 11 seconds), without anaesthetic machine difference. Wash-out was also fresh gas flow dependent and plateaued at 7.5 L/min. **Conclusions**: Circle system wash-in and wash-out show clear fresh gas dependency and varies somewhat between the Aisys and Flow-i. The circle saturation, reaching 1 MAC end-tidal or increasing from 1-1.5 MAC can be achieved with both work-stations within 1.5 minutes at a constant fresh gas flow of 2 and 4 L/min. Wash-out plateaued at 7.5 L/min.

## Introduction

A rapid change in inspired anaesthetic agent is a requisite for the control of the depth of anaesthesia. Low flow anaesthesia has been increasingly adopted, as it is associated with several benefits, including conserving humidity and temperature, which improves the quality of anaesthesia ^i^. Reducing the amount of anaesthetic agent consumed is of interest not only for reducing cost, but also for reducing environmental burden. The merits of reducing flow must not overrule safety, by maintaining adequate oxygen content in the circle, and avoiding hypoxic gas mixture, inadequate anaesthesia control and too light anaesthesia with risk for awareness ^ii, iii^. Increasing and decreasing the circle system anaesthetic concentration during low and minimal flow anaesthesia calls for knowledge around the technique and kinetics of the anaesthetic gas used. We studied wash-in, increase and decrease of the end-tidal gas concentration, within the circle system with two anaesthetic machines, GE Aisys and Maquet FLOW-i, at different fixed fresh gas flow and fixed vaporizer settings in a test model. Our hypothesis was that the wash-in and wash-out should be fresh gas flow dependent, and that the time for reaching target end tidal concentrations would be faster for the FLOW-i device, which does not have a classical reservoir.

## Methods

The two anaesthetic workstations, Aisys (GE Healthcare, Madison, WI, USA) and FLOW-i (Maquet, Solna, Sweden), including a standard CO _2_ absorber and a standard circle system (patient circuit, adult, disposable 1.8 m; GE Healthcare) and a Humid-Vent Filter (Teleflex, Wayne, PA, USA), were connected to a 2 L elastic test reservoir (Intersugical Ltd., East Syracuse, NY, USA).

*Wash-in*: time to reach a stable circle concentration; end-tidal concentration of 1 and 1.5 MAC age adjusted (40 year old male) sevoflurane (2.1 and 3.1%) was studied with the circle connected to the test reservoir. Ventilation was set at tidal volume 500 ml, respiratory rate 10 and PEEP 5 cmH2O, for both devices. The oxygen fraction was set at 0.4. Ventilation settings and FiO2 was kept constant during thire test, wash-in and wash-out- Fresh gas flow was fixed at 300, 500, 1000, 2000 and 4000 ml/minute. The vaporizer setting was fixed at 8% for both devices during the wash-in and 0 at wash-out.

The time to reach 1 MAC and the time to increase from 1-1.5 MAC circle concentration was recorded for each value, based on mean of 3 repeated tests.

*Wash-out*: time to decrease from 1.5 MAC to 0 gas concentration with a fixed fresh gas setting of 2500, 5000, 7500 and 10000 ml/minute.

Workstations at the department of anaesthesia at Danderyds University hospital was used for the study. Gas monitoring was done with each workstations side-stream multi-gas infra-red monitor. These analysers are 0-calibrated automatically at start of the machine. The workstations are further controlled and calibrated in accordance to the service routine of the department.

### Statistics

All data are presented as mean and standard deviation based on 3 repeats. The effects on time events (wash-in: increase from 0 – 1 MAC and further increase up to 1.5 MAC; wash-out: decrease from 1.5 MAC to 0 gas) between fresh gas flows and anaesthetic work-stations were calculated by ANOVA. P<0.05 was considered to be statistically significant. Data was analysed with StatView (v1.04) for MAC.

## Results

Fixed fresh gas flows had a significant impact on the speed of wash-in - time to achieve a stable circle end-tidal sevoflurane concentration of 1 MAC age adjusted (2.1%). The mean time of both machines decreased from 547 ± 83 seconds, at a fixed fresh gas flow of 300 ml/min and fixed vaporizer setting of 8%, to 38 ± 6 seconds at a fresh gas flow of 4000 ml/min. The time to further increase the circle system end-tidal sevoflurane from 1 to 1.5 MAC also showed a significant dependency on fresh gas flow: 330 ± 24 seconds, at a fixed fresh gas flow of 300 ml/min and fixed vaporizer setting of 8%, to 17 ± 11 seconds at a fresh gas flow of 4000 ml/min.

**Figure 1.**
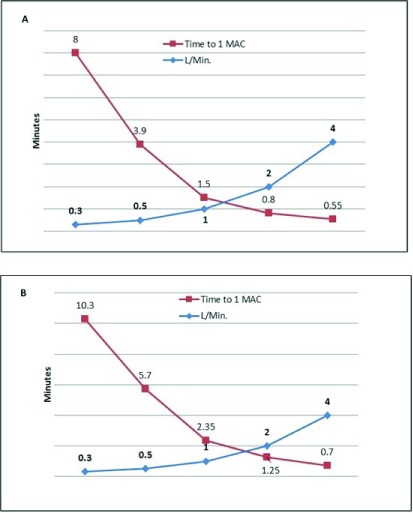
Wash-in time (minutes) to increase to 1 MAC (sevoflurane 2.1%) for ( **A**) Aysis and ( **B**) FLOW-i anaesthetic work stations.

Both anaesthetic work-stations showed the same fresh-gas flow dependent for wash-in and wash-out pattern, but the Aisys showed overall a slightly faster wash-in time ( Table 1 and Figure 1).

**Table 1.** Wash-in time (seconds) to reach 1 and 1.5 MAC circle concentration during constant fresh gas flow and vaporizer setting. Vaporizer set at 8%, tidal volume 500 ml, respiratory rate 10, PEEP 5 and volume controlled ventilation.

	L/min					
	MAC	0.3	0.5	1	2	4
**FLOW-i**	0–1	618	342	141	75	42
	1–1.5	317	191	88	46	25
**Asysis**	0–1	477	232	99	48	33
	1–1.5	344	165	96	22	10

Wash-in (time to reach a stable 1 MAC circle sevoflurane concentration) was achieved within 1.5 minute at a fixed fresh gas flow of 2000 ml/min for both machines tested, 48 ± 2 and 75 ± 2 seconds for the Aisys and Flow-I, respectively (p<0.001), and within 1 minute, mean 33 ± 3 and 42 ± 3 seconds, for Aisys and Flow-i at 4000 ml/min, respectively (p<0.05). A further increase from 1 to 1.5 MAC was achieved within 1 minute for both machines (22 ± 3 and 46 ± 3 seconds for Asysis and Flow-i, respectively) at a fixed fresh gas flow of 2000 ml/min. When a 4000 ml/min was used, the monitoring system was not fast enough to catch the increase for the Aisys, but recorded the increases as 25 ± 10 seconds for Flow-i.

**Figure 2.**
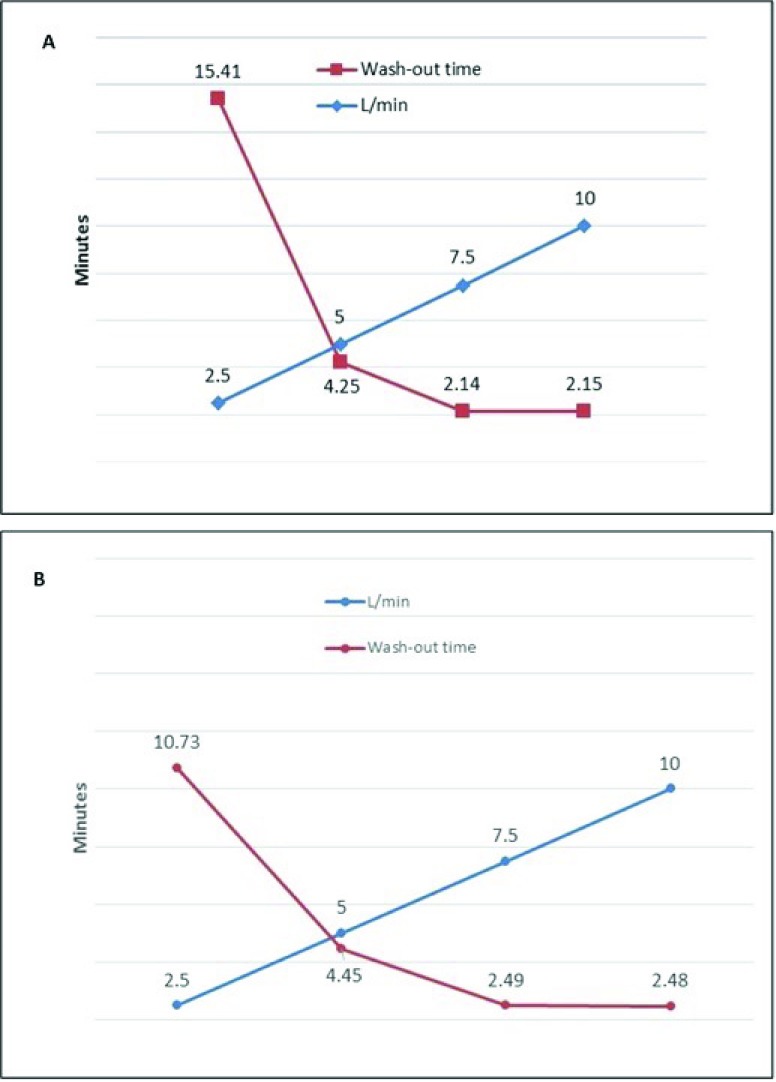
Wash-out time (minutes) for decrease from 1.5 to 0 MAC with sevoflurane at 3.1-0% in ( **A**) Aysis and ( **B**) FLOW-i anaesthetic work stations.

Wash-out was likewise flow dependent, and plateaued at 7.5 L/min ( Table 2 and Figure 2).

**Table 2.** Wash-out time (seconds) to decrease from 1.5 MAC circle concentration with a constant fresh gas flow and closed vaporizer. Tidal volume 500 ml, respiratory rate 10, PEEP 5 and volume controlled ventilation.

	L/min			
	2.5	5	7.5	10
**Aysis**	15.41	4.25	2.14	2.15
**FLOW-i**	10.73	4.45	2.49	2.48

Raw data from the wash-in increase of Et-sevoflurane at fixed fresh gas flow and vaporiser setting Click here for additional data file. Copyright: © 2017 Jakobsson P et al. 2017 Data associated with the article are available under the terms of the Creative Commons Zero "No rights reserved" data waiver (CC0 1.0 Public domain dedication).

Raw data from the wash-out of Et-sevoflurane at zero vaporiser setting and increasing fresh gas flows Click here for additional data file. Copyright: © 2017 Jakobsson P et al. 2017 Data associated with the article are available under the terms of the Creative Commons Zero "No rights reserved" data waiver (CC0 1.0 Public domain dedication).

## Discussion

The present study was set-up to evaluate the impact of fresh gas flow on saturation of and wash-out from the circle system/test-lung set up and whether the modern anaesthesia machines performed differently. We found a clear fresh-gas flow dependency for the time to saturate and wash-out of the circle and test-lung system, as expected. Wash-in to 1 MAC and further increasing the circle concentration to 1.5 MAC decreased with the fresh gas flow, and a 1 and further increase to 1.5 MAC was achieved within 1 minute at a fresh gas flow of 4 L/min. The wash-out was not further improved between 7.5 and 10 L/min fresh gas flow.

We found somewhat surprisingly that the Aisys was slightly faster than the FLOW-i, although the FLOW-i should have a small internal gas reservoir. Lucangelo *et al.* studied the FLOW-i performance regarding tidal volume in case of minor leakage ^iv^. They found the system to be highly accurate. They also described the gas flow control in detail addressing the technical features of the flow regulators. Thus, our hypothesis was a faster saturation of the circle gas with the FLOW-i technology. We cannot give any explicit reason why the wash-in unexpectedly was slower for the FLOW-i. One contributing mechanism may be differences in the vaporizer technology. According to the Maquet user's manual, during controlled ventilation “a larger proportion of the fresh gas is added during the inspiration phase, also contributing to minimizing agent consumption”. The Flow-i vaporiser only injects anaesthetic agent during inspiration and is inactive during the expiratory phase of the cycle which may contribute to the difference in wash-in. The Aisys delivers vapour continuously, like a conventional vaporizer, which might explain why wash-in to a test lung is faster. One should also acknowledge that the gas measurements were done by the built in multi-gas analysers. To what extent that could have impacted the results cannot be stated.

Dosch *et al.* ^v^ studied the change in circle gas composition in three anaesthetic machines and found that fresh gas flow and breathing system volume have the biggest effect on time to equilibrium. In a previous study, we analysed the wash-in of desflurane and sevoflurane during fixed fresh gas flow and vaporizer setting with the Aisys anaesthesia workstation ^vi^. We found, as expected, desflurane to be associated to a significantly faster wash-in compared to sevoflurane with a significant impact from the fresh gas flow: The increase from 0.5 L/min to 1.0 L/min in fresh gas flow reduced the time to reach 1 MAC age adjusted end-tidal concentration from 15.2±2.4 minutes to 6.2±1.3 minutes. We found in that study a rather large variability for sevoflurane, which we considered was related to a combination of circle system gas saturation and uptake.

Kern *et al.* studied the saturation of neonatal anaesthesia systems ^vii^. They found huge differences in the time to reach the end tidal concentration above 95% of inspired. They also found wash-in times to decrease with higher fresh gas flows and higher minute ventilation rates; however, they saw that the effect of doubling fresh gas flow was variable and less than expected. Struys *et al.* made a study much like ours comparing the Zeus apparatus with direct injection of inhaled anaesthetics and the Primus apparatus using a classical out-of-circle vaporizer ^viii^. They found the Zeus to have a faster time course, but their study set-up was different from ours; they used fresh gas and auto control modes, providing a high initial fresh gas bolus. We compared the novel FLOW-i with a similar injection technique and without classical reservoir, and the Aysis with a more classic design. Carette *et al.* studied the performance of the automatic control mode of the FLOW-i ^ix^. The possibility to use an automatic algorithm to reach desired circle, end-tidal concentration is an interesting option and we plan to do further studies assessing the automatic technique. One limitation of the study was that it is an entirely experimental study.

There are several limitations with our study. We studied only wash-in and wash-out in a test lung and indeed there was no uptake of anaesthetic vapour from the breathing circuit and likewise no anaesthetic agent elimination from the blood during wash-out. We used the built in gas monitors. It would have been of value to have had a free-standing well calibrated multi gas sensor. One may also argue that statistical differences seen may not translate into clinical important differences. Further studies assessing the benefits and limitations with the new and costly anaesthetic works stations are warranted.

In conclusion, wash-in, saturation of and wash-out of the circle system is fresh gas flow dependent. A 1 MAC can be reached within 1 minute at a fixed vaporizer setting of 8 at a fresh gas flow of 4 L/min and further increase from 1-1.5 MAC can be reached within 1 minute at a fresh gas flow of 2 L/min. Wash-out was found likewise flow dependent, but the time to reach a zero end-tidal concentration plateaued at 7.5 L/min.

## Ethical statement

This is a test model study. The research does not involve human participants and/or animals, and thus no informed consent has been requested. The set-up is entirely experimental and no human or animals have been exposed to anaesthetics, and thus no ethical review board assessment has been considered necessary.

## Data availability

The data referenced by this article are under copyright with the following copyright statement: Copyright: © 2017 Jakobsson P et al.

Data associated with the article are available under the terms of the Creative Commons Zero "No rights reserved" data waiver (CC0 1.0 Public domain dedication). 

Dataset 1: Raw data from the wash-in increase of Et-sevoflurane at fixed fresh gas flow and vaporiser setting. doi, 10.5256/f1000research.11255.d156064 ^x^

Dataset 2: Raw data from the wash-out of Et-sevoflurane at zero vaporiser setting and increasing fresh gas flow. doi, 10.5256/f1000research.11255.d156065 ^xi^
