# Supplementary material for: Wash-in and wash-out of sevoflurane in a test-lung model: A comparison between Aisys and FLOW-i
Source: F1000Res. 2017 Apr 26;6:389. Originally published 2017 Mar 29. [Version 2] doi: 10.12688/f1000research.11255.2 (PMC5414818; doi:10.12688/f1000research.11255.2)
Supplement: Peer review history

# Peer review: Referee response for version 2

**DOI:** 10.5256/f1000research.12427.r22523

**Published:** 2017-05-05

**Authors:** Thomas Schilling, Moritz A. Kretzschmar

**Affiliations:** 1 Department of Surgical Sciences, Hedenstierna Laboratory, Uppsala University, Uppsala, Sweden; 2 Department of Anaesthesiology and Intensive Care Medicine, Otto-von-Guericke-University Magdeburg, Magdeburg, Germany

In the present small but nice study the authors compared two modern anaesthesia machines (Aisys, GE, Madison, WI, USA, and FLOW-i, Maquet, Solna, Sweden) concerning the time periods of wash-in and wash-out of sevoflurane with different fresh gas flows and constant vaporizer settings in a test lung model. Jakobsson *et al.* can demonstrate that the time to reach 1 MAC (and later on to 1,5 MAC) in the circle systems depends mainly on fresh gas flow.

 In addition, the authors found no major but slight differences between both anaesthetic work-stations. The fact that the Aisys seems to be a little faster than the Flow-i is surprising and cannot be related to the construction type of the respective machine.

 In our opinion, the present experimental study is of importance since optimal flow rates need to be determined and confirmed for both machines.

 We have some comments and questions to the authors:

1. The authors should consider to measure gas concentrations and ventilation mechanics with external, calibrated, standardized devices. The different integrated equipment may be responsible for a major part of the differences.
2. We presume that in both workstations internal test routines to determine leakage had been performed in advance and passed. However, the tolerated leakage of each system should be reported. It should be for the AISYS less than 150ml/min; however, a number for the Flow-i is not defined in its manual. Thus, leakage has to considered different in both machines.
3. In our opinion, the behavior of the gas flow through the gas analyzer may impact the significance of the results. Both machines use about 200ml/min but in the Flow-i the sample gas is returned to the circle system, whereas the AISYS leads the sample obviously to the waste gas.
4. In addition, there are different precision ranges for the vaporizers that are used in both workstations. The precision for the Flow-i ranges from +/- 0,15 ABS% to 0,4 ABS%; The Aladin vaporizer in the AISYS has a precision of +/- 10% between 18-25 °C. At fresh gas flows exceeding 5 L/min and concentrations >5 vol% the manual states that the delivered concentrations fall after a few minutes. The Flow-i is reported to have +/-15% but a temperature range is not given. The question is, were the experiments conducted in these temperature ranges? Here might be a possible explanation for the variability.
5. The manuscript would benefit from a comprehensive revision of typing errors (e.g. “competing interests” paragraph on p. 2.: has received, Pfizer; “Revised” box: “vaporization”; Introduction: “A rapid change in inspired anaesthetic agent...” – “fraction” or “concentration” is missed.)
6. We would like to ask the authors to improve the figures: Please name the X axis for better understanding, please include scatter measurements (SD, SEM).

We have read this submission. We believe that we have an appropriate level of expertise to confirm that it is of an acceptable scientific standard, however we have significant reservations, as outlined above.

# Peer review: Referee response for version 2

**DOI:** 10.5256/f1000research.12427.r22257

**Published:** 2017-05-02

**Authors:** Göran Hedenstierna

**Affiliations:** 1 Hedenstierna Laboratory, Department of Medical Sciences, Clinical Physiology, Uppsala University, Uppsala, Sweden

The authors have made a reasonable revision of their manuscript. I have no further comments.

I have read this submission. I believe that I have an appropriate level of expertise to confirm that it is of an acceptable scientific standard.

# Peer review: Referee response for version 2

**DOI:** 10.5256/f1000research.12427.r22256

**Published:** 2017-04-28

**Authors:** Ian Smith

**Affiliations:** 1 University Hospitals of North Midlands NHS Trust, Stoke-on-Trent, UK

The authors have revised their manuscript to better discuss their observations and speculate on possible causes for these. Some minor typographical errors have also been corrected. While this remains a lab-type study with all of the associated limitations, these have been adequately acknowledged and the paper nevertheless provides a useful starting point for further clinical research and may also prove useful to clinicians who have recently acquired one or other of these workstations

I have read this submission. I believe that I have an appropriate level of expertise to confirm that it is of an acceptable scientific standard.

# Peer review: Referee response for version 1

**DOI:** 10.5256/f1000research.12142.r21842

**Published:** 2017-04-24

**Authors:** Ian Smith

**Affiliations:** 1 University Hospitals of North Midlands NHS Trust, Stoke-on-Trent, UK

The authors have examined the effect of fresh gas flow rates on the wash-in and wash-out of a single volatile anaesthetic agent in two modern anaesthetic workstations under experimental conditions.The investigation is essentially in two parts. The first shows that wash-in and wash-out and flow dependent for both anaesthesia machines. As these are exponential processes, this finding is entirely predictable and rapid wash-in (or wash-out) will only be achieved at fairly high fresh gas flow rates, whatever the internal design of the anaesthetic machine. While it is still important to define optimal flow rates for new equipment, this part of the paper does not really provide any very new information.

 The second part of the study is potentially far more interesting and this is the comparison between the two systems. The authors speculated, quite appropriately in my opinion, that wash-in and wash-out would be faster with the Flow-i system due to its lower internal volume. However, they did not find this to be the case, especially with regard to wash-in where, in fact, the Flow-i was actually the slower of the two systems. What I find very disappointing is that the authors do not really address why they did not obtain the results they anticipated. Although their discussion mentions a few related points, there is almost nothing concerning the differences between the two systems studied or what the implications of these findings might be. The authors also seem not to have commented on the fact that differences between the two machines were not consistent between wash-in and wash-out and whether this provides any insight into the observed results. For me, this would be a far more interesting paper if these differences were explored in a bit more detail. I would also be interested to know if I needed to change the way I altered vapouriser settings and flow rates in practice were I to switch from one of these systems to the other.

 One possible explanation for the apparent differences has already been mentioned in a previous review, namely that there was not really a difference at all, but that using the in-built gas analysers of the machines resulted in apparent differences due to differences in sampling times/mechanisms/algorithms. I also wonder if differences in the vapouriser technology might explain the findings, at least in part. According to the Maquet user's manual, during controlled ventilation “a larger proportion of the fresh gas is added during the inspiration phase, also contributing to minimising agent consumption”. Our technical staff tell me that the Flow-i vapouriser only injects anaesthetic agent during inspiration and is inactive during the expiratory phase of the cycle. If this is correct, and if the Aisys delivers vapour continuously, like a conventional vapouriser, that might explain why wash-in to a test lung is slower than expected. It is also likely under those circumstances that the difference between the machines would be reduced if higher respiratory rates were used and might also be completely different during spontaneous ventilation. Do the authors have detailed knowledge of how the two vapouriser systems function?

 Although the Flow-i, unexpectedly, was slower than the Aisys, this was not consistently observed and again I think this deserves some discussion. For wash-in from 0–1 MAC, the Aisys was consistently faster, with times ranging from 64–79% those of the Flow-i. However, for wash-in from 1–1.5 MAC, the times were much less consistent, ranging from 109% to 40% those of the Flow-i. Neither was any pattern evident for the second stage wash-in, with the Aisys actually being slower at 0.3 and 1 l/min, but faster at 0.5, 2 & 4 l/min. Can the authors explain this at all? Could sampling error be enough to explain the differences?

 The wash-out results are even more confusing. The Flow-i was slower from 5 to 10 litres/minute, but the wash-out times were so fast that the differences are small and probably within the limits of sampling error. However, at 2.5 l/min the Flow-i was 70% faster. This faster wash-out is entirely consistent with the lower internal volume of the Flow-i (as hypothesised by the authors) and, to me, lends support to the concept that the unexpected observations during wash-in are probably related to differences in the function of the vapourisers.

 As the authors state, this was a test model study. However, the results are likely to be used to inform clinical practice. The problem is that the results are unlikely to be reproduced in clinical practice. As the authors used a test lung, there was no uptake of anaesthetic vapour from the breathing circuit. Adding a patient compartment is likely to delay the wash-in of the anaesthetic machine compartment due to uptake of anaesthetic. This should affect both machines to a similar degree, although if the Flow-i preferentially injects vapour during inspiration while the Aisys does not, it is possible that the addition of a patient may affect the systems differently. Wash-out will also be delayed by the addition of anaesthetic vapour to the breathing system from the patient’s lungs. This should affect both systems equally, but in this case will differ with the duration of anaesthesia delivery and hence the amount of agent taken up by the patient. Do the authors have any clinical data to indicate by how much their findings are actually altered in clinical practice?

 A few other comments:

 What determined the number of repeat measurements? In the methods it is stated wash-in times were determined “based on [the] mean of 3 repeated tests”, but in the statistics section it is said data are means “based on 3–5 repeats”.

 It is stated that data are shown as means and standard deviation. However, all of the tables and figures show only mean data. Standard deviations are only given for the data specifically highlighted in the main text. Without standard deviations, the degree of imprecision of the data cannot be assessed.

 Although a statistically significant p value is defined, the authors do not state the minimum size of differences which they considered to be clinically important. In a few cases, the differences are only a few seconds and of little consequences, but in quite a few cases, the differences are 100 seconds or more and represent quite large percentage differences between the systems. An indication of a minimal clinically-important difference, in association with an indication of the degree of variability, would greatly aid interpretation of the results.

 Statistical tests have been applied to the differences in wash-in and wash-out for both systems (combined) at high versus low gas flows, even though these differences are large in magnitude and highly predictable. However, statistical tests do not appear to have been reported for the far more interesting differences between the anaesthesia machines at each flow rate.

I have read this submission. I believe that I have an appropriate level of expertise to confirm that it is of an acceptable scientific standard, however I have significant reservations, as outlined above.

# Peer review: Referee response for version 1

**DOI:** 10.5256/f1000research.12142.r21357

**Published:** 2017-04-13

**Authors:** Göran Hedenstierna

**Affiliations:** 1 Hedenstierna Laboratory, Department of Medical Sciences, Clinical Physiology, Uppsala University, Uppsala, Sweden

This is a straightforward and simple, in a good sense, model study on the wash-in and wash-out of an anesthetic gas in a test lung model. Comparison has been made between two commercially available anesthesia machines, Aisys and Flow-i. A major finding was that with increasing gas flow, wash-in and wash-out times were reduced, as might be anticipated. The difference was small between the two anesthesia machines but there was a slight difference that in itself was unexpected, i.e. the Aisys being a little faster than the Flow-i. The Aisys uses conventional reservoir bag whereas the Flow-i uses an internal reflector that, at least in theory, should reduce any dead space effect and thus shorten time constants in gas dynamics.

 I have some comments.

 Firstly, the wash-in and wash-out times have been measured in a lung model, the same for both anesthesia machines but the gas concentration has been measured with built in equipment in the Aisys and Flow-i and thus different for the two anesthesia machines. At least I interpret of the results this way. This means that there might be a difference in results that are not caused by the different techniques of internal gas reservoir. With different gas analyzers the results may be related to how the analyzers have been calibrated and what algorithms have been used. Ideally, one should use the same gas analyzer for both machines if the intention has been to test the effect of the design of the gas reservoir. This is my major comment and I suggest that the gas analyzers should be compared to an independent reference. Other comments follow below.

 Abstract line 14: 300 *ml/min*.

 Methods, wash in: You might have used additional settings of the ventilator such as tidal volume, respiratory rate and PEEP. At least you might discuss this.

 Results, page 4 line 3 under figure 1: was *not *fast enough.

 Discussion, page 5, third last line in the right column: Why as expected?

 Page 6, left column, second paragraph: Zeus

I have read this submission. I believe that I have an appropriate level of expertise to confirm that it is of an acceptable scientific standard, however I have significant reservations, as outlined above.

# Response

**Published:** 2017-04-13

**Authors:** Jan Jakobsson

**Affiliations:** Department of Physiology and Pharmacology, Karolinska Institutet, Sweden

Dear Referee

**Thank you for comments;**

We acknowledge the limitation using the machine gas monitor. Both machines are equipped with "standard" side-stream multi-gas monitors. These instruments have an internal calibration at start up. We did perform a startup prior to each test. It should also be noticed that we performed the study with standard anaesthetic machines used at our department, machines used for ordinary patient care. We performed the test during late afternoons and evenings. Thus we do expect the IR-multi-gas readings to be adequate.

Abstract line 14: 300 *ml/min*.

There is indeed a text error in the abstract line 14 please excuse, should read 300 ml/min., no doubt

The ventilator settings are presented in the methods section; *The ventilation was set at tidal volume 500 ml, respiratory rate 10 and PEEP 5 cmH2O, for both devices.*

Results, page 4 line 3 under figure 1: much agree, the **was** is indeed missing, please excuse

Discussion, page 5, third last line in the right column: Why as expected?

This is simply because all authors are clinically active and we are so used to see the lower solubility benefit and subsequently faster "blood compartment wash-in" associated to desflurane as compared to sevoflurane. This can indeed be argued when looking merely at circle system equilibration/wash-in. Thank you for most adequat comment.

Page 6, left column, second paragraph: Zeus,

This referes to the Zeus anaesthesia apparatus (Dräger, Lubeck, Germany)

I hope our comments/responses are acceptable and your adequate and effective review.

Best regards

Jan Jakobsson, on behalf of all authors
